# An Internet-Based Psychological Intervention With a Serious Game to Improve Vitality, Psychological and Physical Condition, and Immune Function in Healthy Male Adults: Randomized Controlled Trial

**DOI:** 10.2196/14861

**Published:** 2020-07-24

**Authors:** Lemmy Schakel, Dieuwke S Veldhuijzen, Henriët van Middendorp, Corine Prins, Anne M H F Drittij, Frank Vrieling, Leo G Visser, Tom H M Ottenhoff, Simone A Joosten, Andrea W M Evers

**Affiliations:** 1 Health, Medical and Neuropsychology Unit Institute of Psychology Leiden University Leiden Netherlands; 2 Leiden Institute for Brain and Cognition Leiden University Leiden Netherlands; 3 Department of Infectious Diseases Leiden University Medical Centre Leiden Netherlands; 4 Department of Psychiatry Leiden University Medical Centre Leiden Netherlands

**Keywords:** BCG vaccination, cognitive behavioral therapy, immune system, stress, ICBT

## Abstract

**Background:**

Recently, internet-based cognitive behavioral therapy (ICBT) and serious gaming interventions have been suggested to enhance accessibility to interventions and engagement in psychological interventions that aim to promote health outcomes. Few studies, however, have investigated their effectiveness in the context of simulated real-life challenges.

**Objective:**

We aimed to examine the effectivity of a guided ICBT combined with a serious gaming intervention in improving self-reported psychophysiological and immunological health endpoints in response to psychophysiological and immune-related challenges.

**Methods:**

Sixty-nine healthy men were randomly assigned to the intervention condition, receiving ICBT combined with serious gaming for 6 weeks, or the control condition, receiving no intervention. Self-reported vitality was the primary endpoint. Other self-reported psychophysiological and immunological endpoints were assessed following various challenges, including a bacillus Calmette-Guérin vaccination evoking pro-inflammatory responses, 1 and 4 weeks after the intervention period.

**Results:**

Although the intervention did not affect vitality-associated parameters, self-reported sleep problems (*P*=.027) and bodily sensations (*P*=.042) were lower directly after the intervention compared with controls. Furthermore, wellbeing (*P*=.024) was higher in the intervention group after the psychophysiological challenges. Although no significant group differences were found for the psychophysiological and immunological endpoints, the data provided preliminary support for increased immunoglobulin antibody responses at the follow-up time points (*P*<.05). Differential chemokine endpoints between conditions were observed at the end of the test day.

**Conclusions:**

The present study provides some support for improving health endpoints with an innovative ICBT intervention. Future research should replicate and further extend the present findings by consistently including challenges and a wide range of immune parameters into the study design.

**Trial Registration:**

Nederlands Trial Register NTR5610; https://www.trialregister.nl/trial/5466

## Introduction

Psychological interventions have been shown to be effective in improving self-reported health outcomes [1,2] and immune status [3-5]. Two meta-analytic reviews found modest support for improved immune function after psychological interventions [6,7]. However, it is difficult to draw conclusive findings from these studies due to the large heterogeneity in the incorporated interventions (ie, various types of relaxation, conditioning, disclosure, and stress management interventions) and immunological outcomes (ie, quantitative and qualitative). Moreover, novel developments in psychological treatments may potentially further enhance the effectiveness of psychological interventions in improving self-reported and immunological health outcomes [7]. A rather novel development focuses on providing psychological interventions based on cognitive behavioral therapy (CBT) via the internet (ICBT). CBT focuses on enhancing coping and problem-solving skills and therefore can be applied to improve health management strategies [8]. Furthermore, cognitive behavioral strategies can adjust standards of health and in turn improve quality of life [8]. A meta-analysis showed that the effectiveness of guided ICBT interventions is comparable to face-to-face interventions in patients with chronic somatic conditions [9]. Advantages of ICBT over face-to-face interventions are the increased convenience in use and enhanced flexibility of the specific location and time for users regarding where the intervention sessions are completed [10]. However, adherence rates in ICBT are lower compared to face-to-face interventions; therefore, engagement should be taken into account [11,12].

Engagement can possibly be enhanced by applying persuasive internet-based technologies, such as serious gaming. Serious gaming aims to provide education in an entertaining manner and can therefore be intrinsically motivating [13,14]. A meta-analysis showed evidence for the effectiveness of serious gaming in promoting a healthy lifestyle [15]. Serious games are able to tap into multiple learning processes such as explicit behavior change strategies (eg, goalsetting and transferring knowledge [16]), but also imply more implicit behavior change strategies (eg, priming and evaluative conditioning). Serious gaming could therefore be an interesting technique to add to ICBT interventions to further optimize their effectiveness.

Most research has focused on basal health outcomes; however, health outcomes assessed in situations that challenge actual health status might better represent reactions to real-time stressors [17]. Few studies have incorporated immunological or psychophysiological challenges in their study design. Immunological challenges may comprise in vitro exposure to a chemical substance (eg, lipopolysaccharide or pokeweed mitogen [18,19]) to obtain insights into the cellular responses after a psychological intervention. Immunological challenges can also be applied in vivo to observe subsequent responses (eg, antibody responses after vaccination [20] or healing process of experimentally created wounds [21]). Psychophysiological challenges can provide insights into participants’ responses to stress after a psychological intervention (eg, exposure to a social evaluative stressor). The studies that incorporated challenges into their design often focused on incorporating one specific challenge and did not combine and compare effects on both in vitro and in vivo immunological as well as psychophysiological challenges [17]. For example, a recent systematic review of studies that specifically examined wound healing after a psychological intervention provided some support for the optimization of immunological markers after this challenge [22]. It is possible that incorporating multiple challenges will provide a more concise view on the effectiveness of a psychological intervention and therefore provide further insights into the link between psychological and immunological mechanisms.

The aim of this randomized controlled trial was therefore to investigate whether an ICBT intervention combined with serious gaming to optimize its effectiveness and engagement can effectively improve self-reported psychophysiological and immunological health endpoints in response to in vitro and in vivo immunological and psychophysiological challenges [17,23]. To gather more insights into the potential effectiveness of an ICBT intervention combined with serious gaming intervention in preventing adverse health endpoints and improving immune function, we included healthy participants in this study. Including healthy participants also provided us with the opportunity to incorporate multiple immunological and psychosocial challenges to gather more insights into the mechanisms underlying the effects of psychological interventions on health endpoints. It was hypothesized that participants in the intervention condition would show higher self-reported vitality and related health endpoints after the intervention compared with the control condition. In addition, improved self-reported psychophysiological and immune-related health endpoints after the in vitro and in vivo immunological and psychosocial challenges were expected in the intervention condition compared with the control condition.

## Methods

### Ethical Considerations

The study protocol was approved by the Medical Ethical Committee of Leiden University Medical Centre (registration number P15.099/NL52434.058.15) and preregistered at the Netherlands National Trial Register (NTR5610). The study was conducted in accordance with the Declaration of Helsinki and the International Conference on Harmonisation Guidelines on Good Clinical Practice. Details on the study protocol and design have been published previously [23].

### Study Population

To gather more insights into the potential effectiveness of ICBT combined with serious gaming in preventing adverse health endpoints and improving immune function, we included healthy participants in this study. The inclusion and exclusion criteria are described in detail in a previously published article on the study protocol and design [20]. Briefly, healthy male participants between 18 and 35 years of age without any somatic or psychological conditions interfering with the study protocol were eligible to participate in the study. We only included male participants as the menstrual cycle is known to affect immune function [23,24]. Participants were recruited through digital and printed flyers at various faculties of Dutch universities from February 2016 until April 2018.

### Procedure

Participants were informed that the study was about the effectiveness of a psychological training program directed at optimizing immune function. After signing an informed consent form, participants completed the self-reported and psychophysiological endpoints, and venous blood was collected. Participants who met the inclusion criteria were randomly assigned to a 6-week intervention or control condition. In the week following the 6-week intervention or control period (ranging from 1 to 7 days after completion of the intervention period), all participants again completed the self-reported and psychophysiological endpoints, and blood was collected. Directly afterwards, participants were vaccinated with bacillus Calmette-Guérin (BCG). One day later, a test day followed, on which psychophysiological stress challenges (ie, Paced Auditory Serial Addition Task, Cold Pressor Test, and Trier Social Stress Test) were administered. At the start and end of the test day, self-reported and psychophysiological endpoints were assessed (see Table 1), and blood was again collected. After 4 weeks, a follow-up measurement was conducted, including self-reported endpoints as well as psychophysiological endpoints and collection of a blood sample. Total time investment for participating in the study was around 15-20 hours, depending on the group allocation. This also included 4 visits to the study center. Participants received €200 for their participation.

**Table 1:** Details of the endpoints at each measurement point.

Endpoint	Baseline	After intervention /pre-vaccination	Start test day	End test day	Follow-up
Self-reported endpoints	SVS^a^, CIS-20^b^, RAND-36, PILL^c^, MOS^d^ Sleep, PANAS^e^, and NRS^f^	SVS, CIS-20, RAND-36, PILL, MOS Sleep, PANAS, and NRS	SVS, CIS-20, PANAS, and NRS	PANAS and NRS	SVS, CIS-20, RAND-36, PILL, MOS Sleep, PANAS, and NRS
Psychophysiological endpoints	Heart rate variables, skin conductance, cortisol, and alpha amylase	N/A^g^	Heart rate variables, skin conductance, cortisol, and alpha amylase	Heart rate variables, skin conductance, cortisol, and alpha amylase	Heart rate variables, skin conductance, cortisol, and alpha amylase
Immune endpoints	Unstimulated as well as LPS^h^-stimulated serum samples	Unstimulated as well as LPS-stimulated serum samples	Unstimulated as well as LPS-stimulated serum samples	Unstimulated serum sample	Unstimulated serum sample

^a^SVS: Subjective Vitality Scale.

^b^CIS-20: Checklist Individual Strength.

^c^PILL: Pennebaker Inventory of Limbic Languidness.

^d^MOS: Medical Outcomes Study.

^e^PANAS: Positive and Negative Affect Schedule.

^f^NRS: numeric rating scale.

^g^N/A: not applicable.

^h^LPS: lipopolysaccharide.

### Randomization and Blinding

Participants were randomized to the intervention or control condition based on a 1:1 allocation ratio. A block randomization was performed by the first author with random.org (block size of 4) to control for seasonal influences [23]. The test leader on the test day was blinded for group allocation.

### Intervention

See Table 2 for an overview of the intervention. Participants in the intervention group received a guided ICBT intervention for 6 weeks [23], which was based on an ICBT intervention for chronic somatic diseases developed in our research group [25,26]. The intervention was delivered on the internet platform and software hosted by Karify [27]. The intervention could be accessed individually by the participant. It was provided for free, and the website was password protected. Access was granted by providing participants with an email link in which participants were invited to set up a personal account. The intervention contained 6 modules (goal setting, healthy food and exercise, relaxation, sleep, cognition and worldview, and long-term goals). These modules were guided by a therapist (psychologist supervised by a CBT psychologist) from whom participants received homework assignments and asynchronously provided feedback messages. In addition, participants in the intervention condition played a serious game (ViaNova©) that incorporated comparable modules as the guided intervention (ie, healthy food and exercise, sleep, relaxation, and long-term goals) as part of the ICBT. A subset of these games that focused specifically on food-related health behavior was tested in a previous study that demonstrated preliminary support for the effectiveness of a single serious gaming session in optimizing virtual food choice and implicit food preference [28]. Two weeks after the intervention, participants received a booster session by telephone which lasted 15-30 minutes and focused on relapse prevention by asking participants how they worked on their goals after the last online session and what strategies they used to keep up with their goals. The control condition did not receive any training.

**Table 2:** Overview of the intervention.

Week	Module	Actions
1	Module goal setting	Face-to-face intake with the therapist and setting goals for the online intervention
2	Module healthy food and exercise	Reading information, keeping a diary on goal progress, reading the information provided online by the therapist, completing assignments provided by the therapist
3	Module relaxation	Reading information, keeping a diary on goal progress, reading the information provided online by the therapist, completing assignments provided by the therapist
4	Module sleep	Reading information, keeping a diary on goal progress, reading the information provided online by the therapist, completing assignments provided by the therapist
5	Module cognition and world view	Reading information, keeping a diary on goal progress, reading the information provided online by the therapist, completing assignments provided by the therapist
6	Module long-term goals	Reading information, keeping a diary on goal progress, reading the information provided online by the therapist, completing assignments provided by the therapist

### Challenges

#### In Vitro and In Vivo Immunological Challenges

As an in vitro immunological challenge, heparinized whole blood samples were stimulated in vitro with lipopolysaccharide (LPS) to stimulate cytokine production at baseline (before the intervention), at the start of the vaccination day, and one day later at the start of the test day [23]. The process consisted of stimulating 1 mL of sodium-heparinized blood in BD Vacutainer blood collection tubes (BD, Franklin Lakes, NJ) with LPS (*Escherichia coli*, ultra-pure, Invivogen, Toulouse, France) at a final concentration of 100 ng/mL or as a control without LPS, and samples were incubated at 37 °C for 6 hours. Tubes were spun at 3400 rpm for 10 minutes, and plasma was collected and stored until testing at –80 °C.

In addition, in the week following the intervention (or similar time frame for the control arm), all participants were vaccinated with *Mycobacterium bovis* BCG, a live-attenuated vaccine used against tuberculosis. This vaccine was incorporated as an in vivo challenge to the immune system. BCG (Intervax, via RIVM, Bilthoven, The Netherlands) was administered by intradermal injection (0.1 mL) in the upper arm.

#### Psychophysiological Challenges

On the day post-vaccination, participants were exposed to 3 psychophysiological challenges in the following order: a modified version of the Paced Auditory Serial Addition Task [29], a Cold Pressor Test [30], and the Trier Social Stress Test [31]. All challenges are known to reliably induce psychophysiological stress responses [30-33]. More information regarding these challenges has been published previously [23].

### Primary Endpoints

#### Self-Reported Vitality

The Subjective Vitality Scale (SVS) [34] and Checklist Individual Strength (CIS-20) [35,36] were used to measure self-reported vitality. The composite score of the SVS and CIS-20 was used as a primary endpoint in this study, to gather a rather complete view on vitality. This composite score was determined by subtracting the standardized sum score of the CIS-20 from the standardized sum score of the SVS. Scores on the composite scale can be interpreted as higher scores representing higher self-reported vitality. The SVS and CIS-20 have been shown to be reliable and valid in previous research [37,38] and had good internal reliability in the present study (Cronbach α=.84 and .87, respectively).

### Secondary Endpoints

#### Self-Reported Quality of Life

In addition, the RAND-36 was used to assess physical and mental health-related quality of life by determining sum scores of the subscales physical functioning and emotional wellbeing [39], which has been shown to be reliable and valid in previous literature [40]. Standardized T-scores were computed for both scales, with higher scores representing higher self-reported quality of life.

#### Self-Reported Bodily Sensations

Bodily sensations were measured with the Pennebaker Inventory of Limbic Languidness [41]. The Pennebaker Inventory of Limbic Languidness showed good internal reliability in the present study (Cronbach α=.89).

#### Self-Reported Sleep Problems

Sleep problems were assessed with 9 items of the Medical Outcomes Study Sleep Scale [42], which showed good internal reliability previously [42]. Higher scores on this scale represent lower levels of self-reported sleep problems. Although this questionnaire yielded sufficient internal reliability at follow-up (Cronbach α=.73), the internal reliability in the present study was low at baseline and after the intervention (Cronbach α=.45 and .36, respectively); therefore, the results of this scale in the present study should be interpreted with caution.

#### Self-Reported Wellbeing

Wellbeing was assessed using the 20-item Positive and Negative Affect Schedule [43] and a 7-item numeric rating scale (NRS) on wellbeing. The Positive and Negative Affect Schedule was subdivided into the positive affect scale and the negative affect scale, which both showed good reliability and validity in previous literature [44] as well as good reliability in the present study (Cronbach α =.88 and .70, respectively). On the NRS that was used to measure wellbeing, scores ranged from 0 (not at all) to 10 (very much). Higher scores on this questionnaire represent higher levels of self-reported wellbeing. The NRS showed good internal reliability in the present study (Cronbach α=.80).

#### Psychophysiological Endpoints

Heart rate (HR), heart rate variability (HRV), and skin conductance were assessed continuously with a BIOPAC MP150 system (BIOPAC Systems Inc, Goleta, CA) using AcqKnowledge software version 4.1.1. Furthermore, HR, HRV, and skin conductance were measured at a resting state for 5 minutes at specific time points (see Table 1). Recording of the electrocardiogram signal was performed with an ECG100C module set at 1000Hz. The high pass filter was set at 0.05 Hz and the low pass filter at 35Hz. For HR, electrodes were attached at the sternum and somewhat below the left lower rib. To measure skin conductance, Ag/Agcl electrodes were attached at the medial phalanges of two fingers of the non-dominant hand (ie, the middle and index fingers). A GSR100C module was used to measure skin conductance, set at 1000 Hz. Gain was set at 5 μΩ/V and the low pass filter at 10 Hz. The Physio Data Toolbox Version 0.4 was used for visual inspection of the data as well as for calculating the mean HR, HRV, and skin conductance levels at each time point [45].

Saliva samples were collected to measure cortisol and alpha amylase. Samples were stored at –80 °C until analyzed. Cortisol was assessed in saliva with a competitive electrochemiluminescence immunoassay using a Modular Analytics E602 immunoanalyzer (Roche Diagnostics, Mannheim, Germany). Cortisol activities were measured and expressed in nmol/L. Determination of salivary alpha amylase was performed using a kinetic colorimetric assay for total amylase activity (Cat Nr. 03183742, Roche Diagnostics, Mannheim, Germany) on a routine clinical chemistry analyzer. Amylase activity was measured and expressed in U/L.

#### Immune Endpoints

Blood samples were collected in cloth-activating tubes (BD Vacutainer) at baseline, after the intervention/pre-vaccination, post-vaccination, and at the 4-week follow-up. Samples were clotted for an hour at room temperature before centrifugation at 2500 rcf for 10 minutes, and serum was collected and aliquoted for storage at –80 °C.

The list of cytokines and chemokines that were analyzed is specified in Multimedia Appendix 1. Cytokine and chemokine levels were measured in serum as well as in stimulated or control plasma samples using the multiplex bead array (Bio-Plex Pro Human Chemokine Panel, 40-Plex #171AK99MR2, Bio-Rad laboratories, Veenendaal, The Netherlands [46]). C-reactive protein concentrations were determined in serum by ELISA according to the instructions of the manufacturer (Abnova, Heidelberg, Germany) at baseline, at the start of the vaccination day, at the start of the test day, and at follow-up.

In addition, immunoglobulin G (IgG) antibody levels were evaluated at baseline and 4 weeks after vaccination. Purified protein derivative (5 μg/mL; Statens Serum Institute, Copenhagen, Denmark) was coated to 96 well Microlon plates (Greiner, Alphen aan den Rijn, The Netherlands). Sera were diluted 1 to 25 and incubated overnight. IgG antibody binding was detected using HRP-labelled polyclonal rabbit anti-human IgG (Dako, Glostrup, Denmark), staining with TMB substrate buffer (Sigma Aldrich, Zwijndrecht, The Netherlands), stopped with H2SO4 and OD450 reading [47].

### Statistical Analyses

All analyses were performed using SPSS Statistics (version 25; IBM Corp, Armonk, NY). As described in our design paper [23], a total sample size of 60 participants was deemed sufficient to detect scientifically and clinically relevant differences in the incorporated primary endpoint. An analysis of covariance (ANCOVA) with condition (intervention vs control) as the between-subjects factor, vitality after the intervention as the dependent variable, and baseline vitality as the covariate was conducted to assess the primary hypothesis that participants in the intervention condition would show higher self-reported vitality after the intervention (pre-vaccination) compared with the control condition. In addition, when a significant effect was found in the ANCOVA, it was investigated whether the effects were also present at the other time points. This was done with repeated measures analysis of variance (ANOVA) with condition (intervention vs control) as the between-subjects factor and time (ie, baseline, after intervention/pre-vaccination, after vaccination, follow-up) as the within-subjects factor. For the repeated measures ANOVAs, we were specifically interested in the interaction effects between time and condition, as well as in the main effects of time, which are therefore specified in the Results section. To examine at which time point(s) groups differed on vitality, represented by a significant interaction effect between time and condition in the repeated measures ANOVA, Holm’s corrected ANOVAs were performed to compare the intervention condition with the control condition at specific time intervals by calculating difference scores between baseline and each of the other time points. Since we did not observe substantial missing data or deviations from the actual timeline within participants, we decided to test the secondary endpoints in a similar way (repeated measures ANOVA) as for the primary endpoint instead of the preplanned multilevel analyses for the secondary endpoints [23]. The results for bodily sensations, quality of life, and sleep problems were analyzed as described at 3 time points (ie, baseline, after intervention/pre-vaccination, follow-up). As the items on these questionnaires were based on the experiences of the last 4 weeks, these questionnaires were not completed post-vaccination.

To test any group differences for wellbeing and positive and negative affect in response to the test day, repeated measures ANOVAs were performed for wellbeing and positive and negative affect with condition (intervention vs control) as the between-subjects factor and the 4 time points (ie, baseline, start of the test day, end of the test day, follow-up) as the within-subjects factor. Data on cortisol, alpha amylase, HR, HRV, and skin conductance were analyzed in a similar way.

For both serum and LPS whole blood stimulation assay, principal component analysis was performed to identify and subsequently exclude extreme outliers. Interleukin (IL)-6 and IL-8 were excluded from the LPS whole blood stimulation analysis. For each time point comparison, two types of linear models were fitted. The first was a linear multiple regression model using Δ-cytokine concentrations at different time points (ie, pg/mL at start of the test day – pre-vaccination, pg/mL at the end of the test day – pre-vaccination, and pg/mL at follow-up – baseline) as dependent variables to estimate the effect of the intervention as an independent variable on changes in cytokine concentrations while correcting for age. The second was a linear mixed model with a random intercept per subject to estimate the effect of time on cytokine levels in either the control or intervention group while correcting for age. Resulting *P* values were false discovery rate (FDR)–corrected to obtain *q* values. Data were mean-centered and scaled to standard deviation units for the generation of volcano plots. Finally, principal component analysis, fitting of multiple linear regression models and linear mixed models, and plotting of analysis results were performed using R version 3.5.0 with the following packages: ‘mixOmics’ [48], ‘lme4’ [49], ‘lmerTest’ [50], and ‘ggplot2’ [51].

## Results

### Sample Characteristics

**Figure 1:**
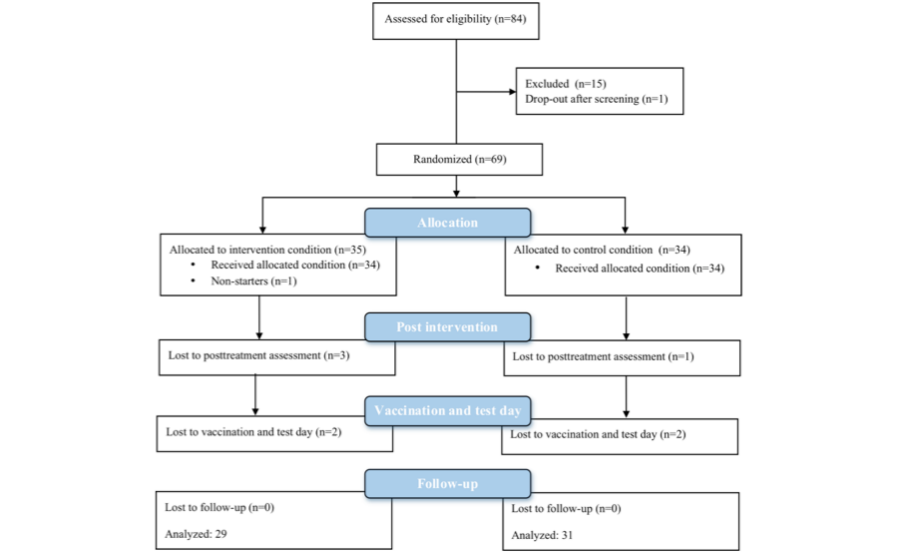
Flow diagram.

Of the 84 participants assessed for eligibility, 14 participants did not meet the inclusion criteria (7 due to somatic morbidity, 5 due to psychological morbidity, and 2 due to practical planning issues). One participant dropped out of the study directly after the screening. Therefore, 69 participants were randomized to one of the two conditions in the present study (see Figure 1). Then, 3 participants dropped out of the study, 1 in the control condition and 2 in the intervention condition. Additionally, 1 participant did not start in the intervention condition after group allocation, due to time constraints. Due to global production problems of the BCG vaccine, 2 participants in the intervention condition and 2 participants in the control condition dropped out of the study after completion of the primary endpoint. Furthermore, 1 participant in the intervention condition dropped out of the study after completion of the intervention due to time constraints. This resulted in 31 participants in the control condition and 29 participants in the intervention condition that completed all visits. Analyses were performed for available data. No significant differences were found in age or BMI between the participants in the control and intervention conditions (*P*>.05). See Table 3 for an overview of the age, BMI, and baseline level of vitality of the participants.

### Primary Endpoint

#### Vitality

**Figure 2:**
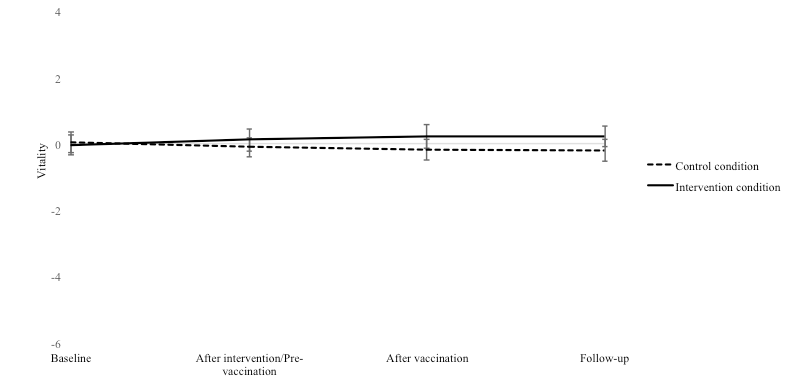
Mean and standard error of self-reported vitality at baseline, after intervention (pre-vaccination), after vaccination, and at follow-up, separately for the control condition and the intervention condition.

No significant differences were found between the groups for self-reported vitality within 1 week after the intervention (pre-vaccination; *P*=.43). The descriptive results for vitality at all time points are displayed in Figure 2.

**Table 3:** Demographic characteristics for the control and intervention conditions.

Characteristics	Control condition	Intervention condition
Age (years), mean (SD)	22.9 (4.1)	22.5 (2.3)
BMI (kg/m^2^), mean (SD)	23.0 (2.8)	22.5 (2.4)
Standardized vitality score, mean (SD)	0.05 (1.84)	–0.05 (1.66)

### Secondary Endpoints

#### Self-Reported Quality of Life, Bodily Sensations, Sleep, Positive and Negative Affect, and Wellbeing

In Multimedia Appendix 2, the results for quality of life for the physical (A) and mental (B) quality of life subscales are shown. Both ANCOVAs did not yield any significant group differences (*P*=.92, *P*=.24, respectively).

**Figure 3:**
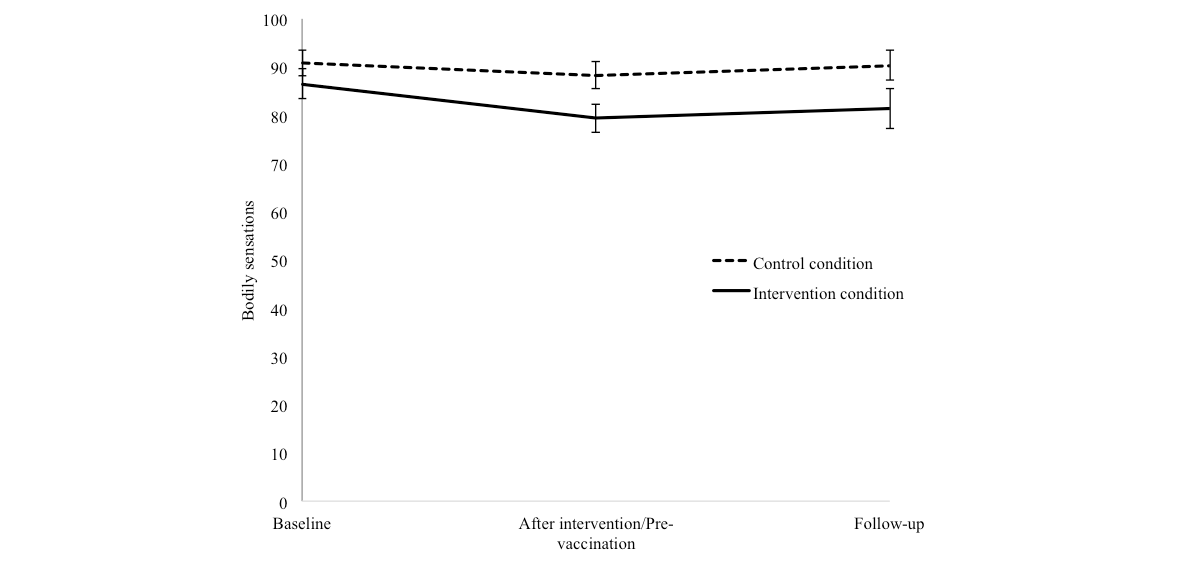
Mean and standard error of the mean of self-reported bodily sensations at baseline, after intervention (pre-vaccination), and at follow-up, separately for the control condition and intervention condition.

Figure 3 depicts the results for bodily sensations. The ANCOVA yielded a significant main effect for condition (*F*_1,62_=4.30, *P*=.042, *n^2^* =.56), indicating fewer bodily sensations for the intervention condition compared with the control condition directly after the intervention (pre-vaccination). The repeated measures ANOVA yielded a significant main effect of time (*F*_1.65,79.03_=7.30, *P*=.002). Irrespective of condition, Holms corrected pairwise comparisons showed a significant decrease from baseline to after the intervention (pre-vaccination; t_64_=3.16, adjusted *P*=.004), as well as a significant decrease from baseline to follow-up (t_49_=2.43, adjusted *P*=.019). No significant interaction effect between time and condition was found (*P*=.36).

**Figure 4:**
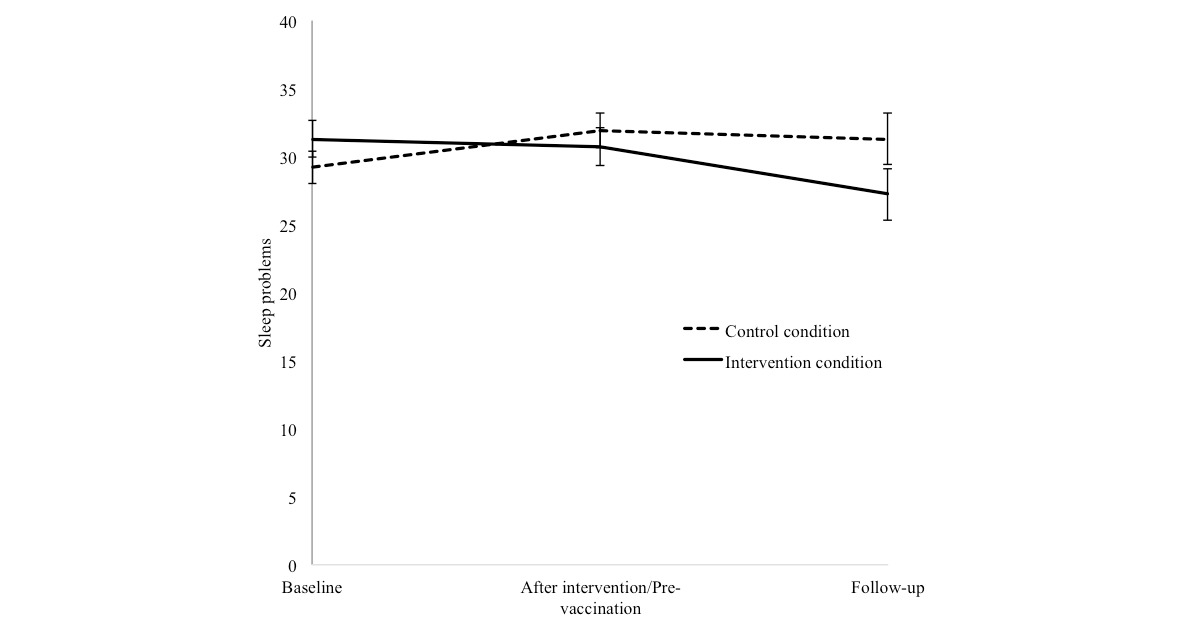
Mean and standard error of the mean of sleep problems at baseline, after intervention (pre-vaccination), and at follow-up, separately for the control condition and the intervention condition.

The results for sleep problems are presented in Figure 4. The ANCOVA showed a trend for an effect of the intervention (*F*_1,62_= 3.30, *P*=.074, *n^2^*=.44). The repeated measures ANOVA did not yield a significant effect of time (*P*=.18) but showed a significant interaction between time and intervention (*F*_1.66,104.74_=4.02, *P*=.027, *n^2^*=.06). Holms corrected pairwise comparisons showed a significant difference between the intervention condition and control condition from baseline to after the intervention (pre-vaccination; *F*_1,63_=4.60, adjusted *P*=.036, *n^2^*=.07), as well as from baseline to follow-up (*F*_1,63_=6.23, adjusted *P*=.030, *n^2^*=.09), indicating fewer sleep problems directly after the intervention (pre-vaccination) and also at follow-up for the intervention condition compared with the control condition.

The results for positive and negative affect are shown in Multimedia Appendix 3A and Multimedia Appendix 3B, respectively. For positive affect, no significant interaction effect between time and condition was found, *P*=.69. Negative affect also yielded no significant interaction between time and condition, *P*=.15.

**Figure 5:**
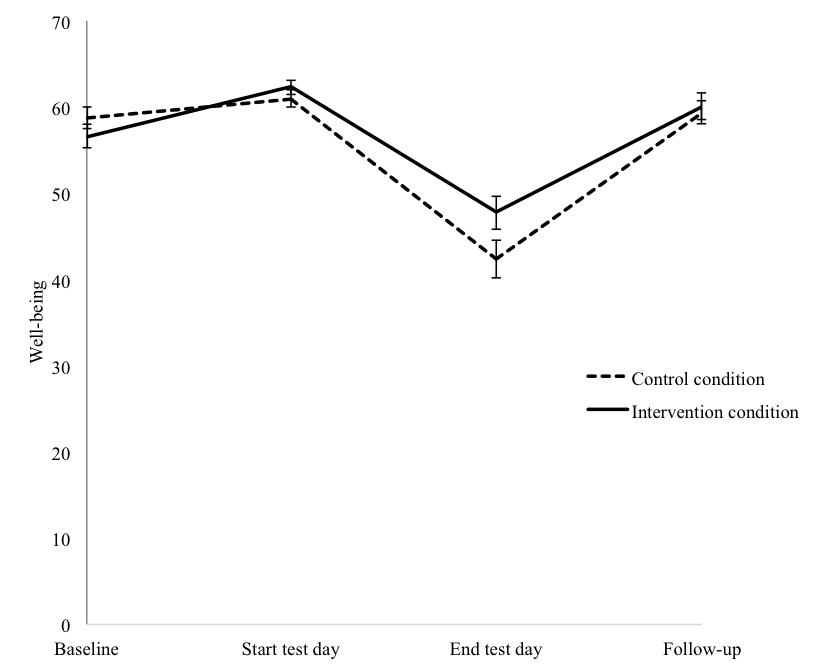
Mean and standard error of the mean of self-reported wellbeing at baseline, the start of the test day, the end of the test day, and at follow-up, separately for the control condition and intervention condition.

For wellbeing, the results are shown in Figure 5. The repeated measures ANOVA yielded a significant main effect of time (*F*_2.38,138.04_=18.97, *P*<.001) and a significant interaction effect between time and intervention (*F*_2.38,138.04_= 3.27, *P*=.033, *n^2^*=.14). Holms corrected pairwise comparisons showed a significant difference between the intervention condition and control condition from baseline to the end of the test day (*F*_1,58_=7.45, adjusted *P*=.024, *n^2^*=.11), indicating less of a decrease in self-reported wellbeing from baseline to the end of the test day for the intervention compared with the control condition.

#### Psychophysiological Endpoints

Multimedia Appendix 4 shows the descriptive statistics for HR, skin conductance, HRV, cortisol, and alpha amylase for the control and intervention groups. For cortisol, the repeated measures ANOVA yielded no significant interaction between time and condition (*P*=.11). Similar results were found for alpha amylase, as no significant interaction effect between time and condition was found (*P*=.90).

For HR, a significant main effect of time was found (*F*_2.40,132.24_=11.37, *P*<.001). Irrespective of the conditions, Holms corrected pairwise comparisons showed a significant decrease from baseline to the end of the test day (t_56_=–3.78, adjusted *P*<.001). A trend was found for an interaction effect between time and condition (*F*_2.40,132.24_=2.44, *P*=.081), indicating a lower HR at follow-up in the intervention condition compared with the control condition. For HRV, a significant main effect of time was found (*F*_1.49,80.29_=4.74, *P*=.019), which varied over time. Holms corrected pairwise comparisons indicated no significant differences over time. No significant interaction effect was found between time and condition (*P*=.15). For skin conductance, no significant main effect of time (*P*=.46) nor an interaction effect between time and condition was found (*P*=.26).

#### Immune Endpoints

**Figure 6:**
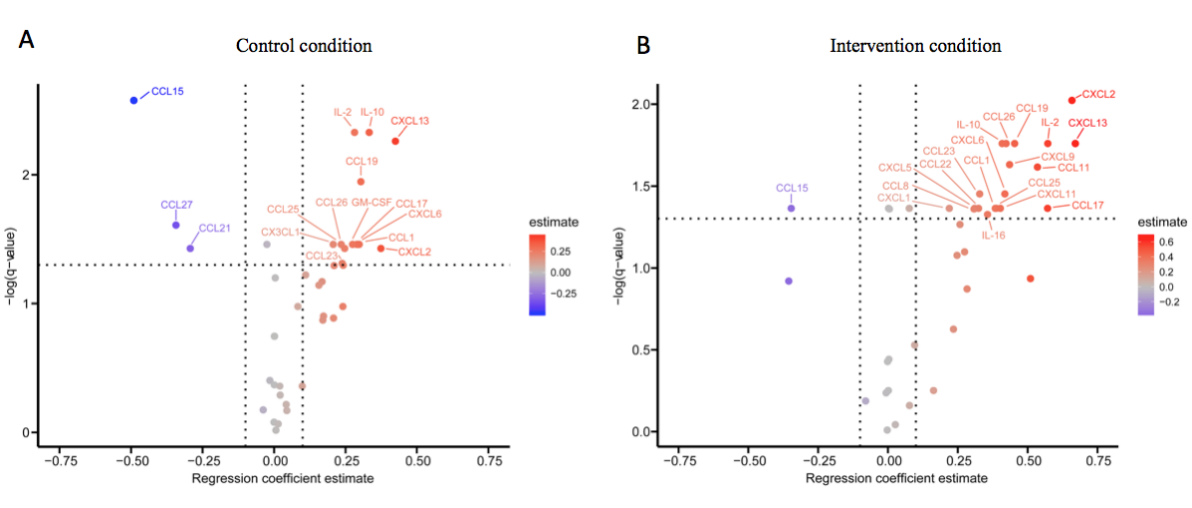
Volcano plots for the comparison between pre-vaccination to the end of the test day for the (A) control and (B) intervention conditions. Significance is displayed on the y axis, and estimate of variance is displayed on the x axis.

Figure 6 shows the volcano plots of significantly upregulated and downregulated serum analytes between pre-vaccination to the end of the test day. Negative values indicate analytes that are downregulated at the end of the test day compared with pre-vaccination, and positive values indicate upregulated analytes at the end of the test day compared to pre-vaccination. Analytes with an estimated effect <0.1 were not considered, since those estimates frequently represent very small changes in cytokine levels below the detection limits of variation in technical duplicates.

The multivariate linear regressions yielded no significant differences between the intervention and control groups at any time point. We therefore exploratively investigated the kinetic changes of the control and intervention conditions individually. Within the control or intervention group, significant changes over time were identified for unique sets of analytes. For the control condition, significant increases for various cytokines and chemokines (ie, IL-2, IL-10, chemokine [C-C motif] ligand [CCL]1, CCL17, CCL19, CCL23, CCL25, CCL26, chemokine [C-X-C motif] ligand [CXCL]2, CXCL6, CXCL13, CX3CL1, granulocyte-macrophage colony-stimulating factor) as well as significant decreases in other chemokines (ie, CCL2, CCL15, CCL21, CCL27; all FDR-corrected *P*<.05) between pre-vaccination and end of the test day were found. For the intervention condition, significant increases were also found for various cytokines and chemokines from pre-vaccination to end of the test day (ie, IL-1β, IL-2, IL-8, IL-10, IL-16, CCL1, CCL8, CCL11, CCL17, CCL19, CCL22, CCL23, CCL25, CCL26, CXCL1, CXCL2, CXCL5, CXCL6, CXCL9, CXCL11, CXCL13, macrophage migration inhibitory factor, tumor necrosis factor [TNF]-α) and a significant decrease in CCL15 (all FDR-corrected *P*<.05). The results for upregulated IL-8, CXCL5, and TNF-α as well as for downregulated CCL15 are shown in Multimedia Appendix 5, as these analytes showed the most prominent group differences. Similar results were found from the start of the test day to the end of the test day. No significant differences were found from baseline to follow-up in the control condition, although the intervention condition showed significant increases in serum IL-10, CCL19, and CXCL9 concentrations as well as a significant decrease in CCL15 (all FDR-corrected *P*<.05).

The results for the IgG antibody levels are displayed in Multimedia Appendix 6. The multivariate linear regressions yielded no significant differences between the intervention and control groups at any time point for IgG antibody levels. When looking at the within-group changes over time separately for the intervention and control conditions, no significant differences were found from baseline to follow-up in the control condition, whereas the intervention condition showed significant increases in purified protein derivative–specific IgG levels (FDR-corrected *P*<.05).

Serum C-reactive protein levels were not significantly different between groups (data not shown).

LPS stimulation of whole blood samples did not induce significant differences between the intervention and control groups (all FDR-corrected *P*>.05). In an explorative analysis, we investigated the intervention condition and control condition separately for the different time ranges. For the control condition from baseline to the test day, we found significant increases in IL-1β and TNF-α (both FDR-corrected *P*<.05), whereas no significant differences were found for the intervention condition.

## Discussion

### Overview

The aim of the present study was to investigate the effects of an ICBT combined with a serious gaming intervention on optimizing self-reported psychophysiological and immunological endpoints in response to in vitro and in vivo immunological and psychophysiological challenges. No significant differences between the intervention and control conditions were found for self-reported vitality. The intervention group did show fewer bodily sensations and fewer sleep problems after the intervention. Furthermore, the intervention group had higher self-reported wellbeing after different psychophysiological stressors compared with the control group. No significant group differences were found for the psychophysiological and immunological endpoints, although some preliminary support was found for improved outcomes on HR variables as well as increased IgG antibody responses at follow-up and differential chemokine endpoints at the end of the test day in the intervention compared with the control condition. The present study thus provides a first step towards unraveling the effectiveness of an electronic health psychological intervention combined with serious gaming elements on optimizing various self-reported psychophysiological and immunological health endpoints.

### Primary Findings

Although the intervention condition showed improved self-reported vitality and the control condition did not, no significant group differences were found. Also, no significant group differences were found for quality of life; however, these scores were already rather high at baseline for both groups. We included a healthy population, which presumably already possessed a good quality of life that could not be maximized further by our psychological intervention. In contrast, bodily sensations, including headache, itch, and other negative sensations, and sleep problems were significantly decreased after the intervention, compared with the control condition. This is in line with previous studies that showed that ICBT can decrease sleep problems and headache symptoms in patients with insomnia or chronic headache [52-55]. As bodily sensations and sleep problems affect general health outcomes [56,57], the intervention was effective in optimizing precursors of health. Due to the variations in the findings for bodily sensations, sleep problems, quality of life, and vitality, however, no conclusive view on the effectiveness of the intervention in improving self-reported health endpoints can be formulated. Furthermore, as the internal reliability of the Medical Outcomes Study Sleep Scale was insufficient in the present study, the outcomes on sleep should be interpreted with caution. However, possible health benefits may become especially clear when the system is challenged. The present study therefore also investigated the results of self-reported endpoints in response to in vitro and in vivo immunological as well as psychophysiological challenges. Although no significant differences were found between conditions in positive and negative affect, a higher self-reported wellbeing was found at the end of the test day for the intervention condition compared with the control condition. It is possible that the healthy population included here already possessed sufficient resilience and skills to handle the immunological and psychophysiological challenges. Future studies should therefore also include participants at risk for health problems, including participants with chronic somatic conditions or with (sub)clinical levels of anxiety or depression to see whether they may benefit from such a psychological intervention [58].

### Secondary Findings

When specifically assessing the psychophysiological health endpoints (ie, HR, HRV, skin conductance, cortisol, and alpha amylase), preliminary evidence for improved endpoints after the intervention was found. Particularly, the intervention condition had a lower HR at follow-up as compared with the control condition. Although not significant, the results for HRV showed a similar pattern. As lower HR and higher HRV can be seen as biomarkers for better stress-related health outcomes [59-61], these data cautiously support the effectiveness of the psychological intervention on optimizing health. However, no significant effects were found for skin conductance, cortisol, and alpha amylase. The results therefore provide limited support for optimizing the response of the sympathetic-adrenal-medullar axis, but no support for influencing the hypothalamic-pituitary-adrenal axis, whereas the sympathetic-adrenal-medullar axis and hypothalamic-pituitary-adrenal axis are known to interact with each other in order to keep allostasis [62]. In addition, as no group differences were found on the test day for HR, HRV, cortisol, and alpha amylase, more research is needed on the external validity and clinical relevance of the present findings on psychophysiological health endpoints.

For the immune endpoints, the between-group analyses yielded no significant findings. The explorative analyses showed significant alterations in several cytokines and chemokines from baseline to follow-up in the intervention condition, whereas no significant alterations were found in the control condition between these time points, providing some cautious support for higher responses for most analytes at the follow-up in the intervention condition. Previous literature on the effectiveness of psychological interventions on optimizing immune function have not yet focused specifically on cytokines and chemokines [6]. Cytokines and chemokines are known to have a significant influence on inflammatory processes, as they provide directional cues for the movement and tissue homing of leukocytes [63,64]. To make more conclusive statements on the effectiveness of psychological interventions in optimizing chemokine functioning, future research should incorporate a wide range of analytes with varying immunological characteristics into the study design, in order to replicate the present findings and to gather more insights in the mechanisms underlying differential immune responses after a psychological intervention.

Concerning the in vivo challenge (ie, the BCG vaccination), we found increased IgG antibody levels from baseline to follow-up for the intervention condition, whereas no such significant differences were observed in the control condition. This finding provides some preliminary support for an altered host response to the BCG vaccine after the intervention. This preliminary finding is in line with a previous study from Petrie et al [20] who found higher antibody levels in response to a hepatitis B vaccine in the intervention condition receiving an emotional disclosure intervention compared to a control condition receiving no intervention. In contrast to a hepatitis B vaccine, the BCG vaccine, being a live vaccine, actually is a human challenge model and as such approximates immune responses that are observed after natural infections [23]. Since antibody titers in the present study were not different in the between-group analyses, the findings need to be interpreted with caution. The present study was the first to incorporate BCG vaccination, and future studies incorporating BCG into the study design should provide further insights into the effects of training towards this infectious challenge.

When looking at the in vitro immunological challenge, the between-group analyses on LPS-stimulated cytokines and chemokines yielded no significant differences. In exploratory analyses, we found that IL-1β, IL-8, CXCL5, and TNF-α were significantly increased from pre-vaccination to the start of the test day in the intervention but not in the control group. Furthermore, CCL2, CCL21, and CCL27 were significantly decreased from pre-vaccination to end of the test day only in the control group but not in the intervention group. Those findings suggest differential immune activation between the groups. However, the data do not support altered immune function following a psychological intervention in response to LPS as an in vitro immunological challenge. Moreover, LPS is a rather strong immune activator, possibly having masked subtle immunologic differences between the intervention and control groups.

### Limitations

Despite the innovative features of the present study (ie, the combination of innovative intervention components directed at both automatic and conscious information processing and behavior change, multiple in vitro and in vivo immunological and psychophysiological challenges, as well as the inclusion of a wide range of self-reported and psychophysiological endpoints), it has some limitations that should be mentioned. First, the present study population consisted of healthy men between 18 and 35 years of age. This represents a homogeneous healthy sample; however, future research should investigate whether the intervention might be (more) effective in other (at-risk) populations. Second, the present study design does not allow us to unravel the effectiveness of separate intervention components or separate challenges. Previously, preliminary support for the effectiveness of serious games on virtual food choice and implicit food preference was found [65]. Future research could further investigate the add-on effectiveness of serious games in optimizing engagement with the intervention and subsequent health endpoints by comparing ICBT with serious games versus ICBT alone. Moreover, future studies may investigate the effectiveness of serious games for adherence to the ICBT treatment. Third, although we tried to keep track of the time participants spent on the serious game by saving log files of the gaming activity, those log files were saved offline by participants themselves, and we did not receive log files from each participant, meaning that we could not verify whether they actually played the game 5 days a week. Although the therapist that guided the intervention tried to keep track of the gaming frequency by asking participants to report on their gaming activities in the online electronic health intervention, future studies should attempt to receive live tracking via online electronic records. Fourth, although we blinded the test leader to group allocation on the test day, the test leaders for the other measurement points were not blinded to group allocation. Although we do not have any indications that this has influenced our results, this cannot be excluded. Finally, although we asked participants not to use drugs and alcohol 48 hours before each measurement and we checked this by verbally asking them whether they used alcohol or drugs, we cannot be entirely sure that participants did not violate these rules. As consumption of alcohol and drugs can alter cytokine responses [66], future research should include quantification of alcohol and drug consumption with objective tests.

### Conclusion

In conclusion, although the present study did not find support for the optimization of vitality, it did find some support for the effectiveness of an ICBT combined with a serious gaming intervention in decreasing bodily sensations and sleep problems. Also, the present study showed that the intervention participants had higher levels of self-reported wellbeing in response to the psychophysiological challenges than control participants. Additionally, specific IgG antibody levels were increased at 4 weeks after BCG vaccination in the intervention condition. As this is one of the first studies incorporating multiple challenges to evaluate the effects of a psychological intervention on health endpoints, the present study provides a first step towards improving health endpoints with a psychological intervention, although clearly more research is needed on this topic. Future research should further investigate whether tailoring the intervention to specific populations, including patients with chronic somatic conditions or participants with (sub)clinical levels of stress or anxiety issues, enhances efficacy and impacts relevant disease-related parameters and biomarkers. Given the innovative study design, combining multiple new elements, future studies should consistently incorporate challenges and a wide range of immune parameters into the study design in order to get a more complete view on the effects of innovative psychological interventions.

## Appendix

Multimedia Appendix 1 Overview of chemokines and other cytokines that were analyzed in the 40-plex assay.

Multimedia Appendix 2 Mean and standard error of the mean of self-reported physical quality of life (A) and mental quality of life (B) T-scores at baseline, after intervention (pre-vaccination), and at follow-up, separately for the control condition and the intervention condition. A higher score on the y-axis represents a higher quality of life.

Multimedia Appendix 3 Mean and standard error of the mean of the standardized scores for self-reported positive affect (A) and negative affect (B) at baseline, the start of the test day, the end of the test day, and at follow-up, separately for the control condition and the intervention condition. A higher score on the y-axis represents a higher level of self-reported positive affect and negative affect, respectively.

Multimedia Appendix 4 Means and standard deviations for heart rate, skin conductance, heart rate variability, as well as cortisol and alpha amylase, separately for the control condition and the intervention condition.

Multimedia Appendix 5 Boxplots for the upregulated IL-8 (A), CXCL5 (B) and TNF- α (C), as well as for the downregulated CCL15 (D) for the control condition (left graph) and intervention condition (right graph) separately at baseline, pre-vaccination, start test day, end test day and follow-up. A higher level in pg/ml on the y-axis represents a higher cytokine/chemokine level.

Multimedia Appendix 6 Boxplots with the OD450 readings for the control condition (left graph) and intervention condition (right graph) separately with the PPD specific IgG antibody levels at baseline and follow-up. A higher OD450 reading on the y-axis represents a higher IgG antibody level.

Multimedia Appendix 7 CONSORT‐eHEALTH checklist (V 1.6.1).

## Abbreviations

ANCOVA: analysis of covariance.
ANOVA: analysis of variance.
BCG: bacillus Calmette-Guérin.
CBT: cognitive behavioral therapy.
CCL: chemokine (C-C motif) ligand.
CIS-20: Checklist Individual Strength.
CXCL: chemokine (C-X-C motif) ligand.
FDR: false discovery rate.
HR: heart rate.
HRV: heart rate variability.
ICBT: internet-based cognitive behavioral therapy.
IgG: immunoglobulin G.
IL: interleukin.
LPS: lipopolysaccharide.
MOS Sleep: Medical Outcomes Study Sleep Scale.
NRS: numeric rating scale.
PANAS: Positive and Negative Affect Schedule.
PILL: Pennebaker Inventory of Limbic Languidness.
SVS: Subjective Vitality Scale.
TNF: tumor necrosis factor.
