# Supplementary material for: An Internet-Based Psychological Intervention With a Serious Game to Improve Vitality, Psychological and Physical Condition, and Immune Function in Healthy Male Adults: Randomized Controlled Trial
Source: J Med Internet Res. 2020 Jul 24;22(7):e14861. doi: 10.2196/14861 (PMC7414409; doi:10.2196/14861)
Supplement: Multimedia Appendix 1 [file jmir_v22i7e14861_app1.docx]

**Multimedia Appendix 1.** Overview of chemokines and other cytokines that were analyzed in the 40-plex assay.

| IL-1β | IL-2 | IL-4 | IL-6 | IL-8 | IL-10 | IL-16 | IP-10 |
| --- | --- | --- | --- | --- | --- | --- | --- |
| CCL1 | CCL2 | CCL3 | CCL7 | CCL8 | CCL11 | CCL13 | CCL15 |
| CCL17 | CCL19 | CCL20 | CCL21 | CCL22 | CCL23 | CCL24 | CCL25 |
| CCL26 | CCL27 | CXCL1 | CXCL2 | CXCL5 | CXCL6 | CXCL9 | CXCL11 |
| CXCL12 | CXCL13 | CXCL16 | CX3CL1 | GM-CSF | MIF | TNF-α | IFN-γ |
