# Supplementary figures and images for: An Internet-Based Psychological Intervention With a Serious Game to Improve Vitality, Psychological and Physical Condition, and Immune Function in Healthy Male Adults: Randomized Controlled Trial
Source: J Med Internet Res. 2020 Jul 24;22(7):e14861. doi: 10.2196/14861 (PMC7414409; doi:10.2196/14861)

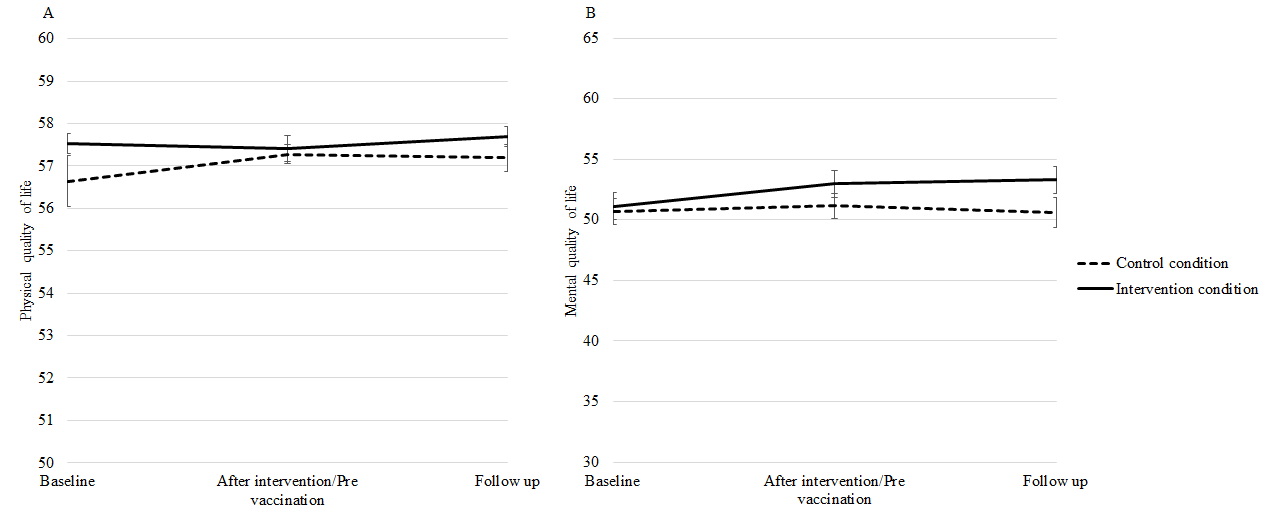

Supplement: Multimedia Appendix 2 [file jmir_v22i7e14861_app2.png]

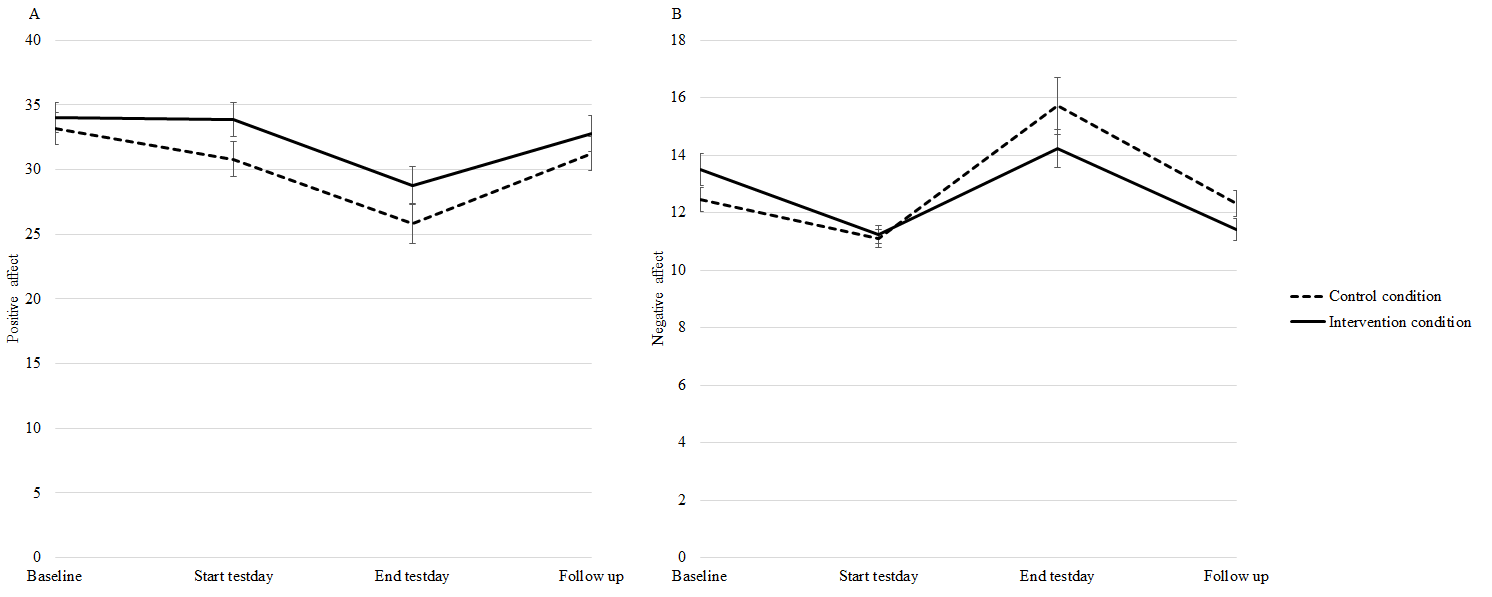

Supplement: Multimedia Appendix 3 [file jmir_v22i7e14861_app3.png]

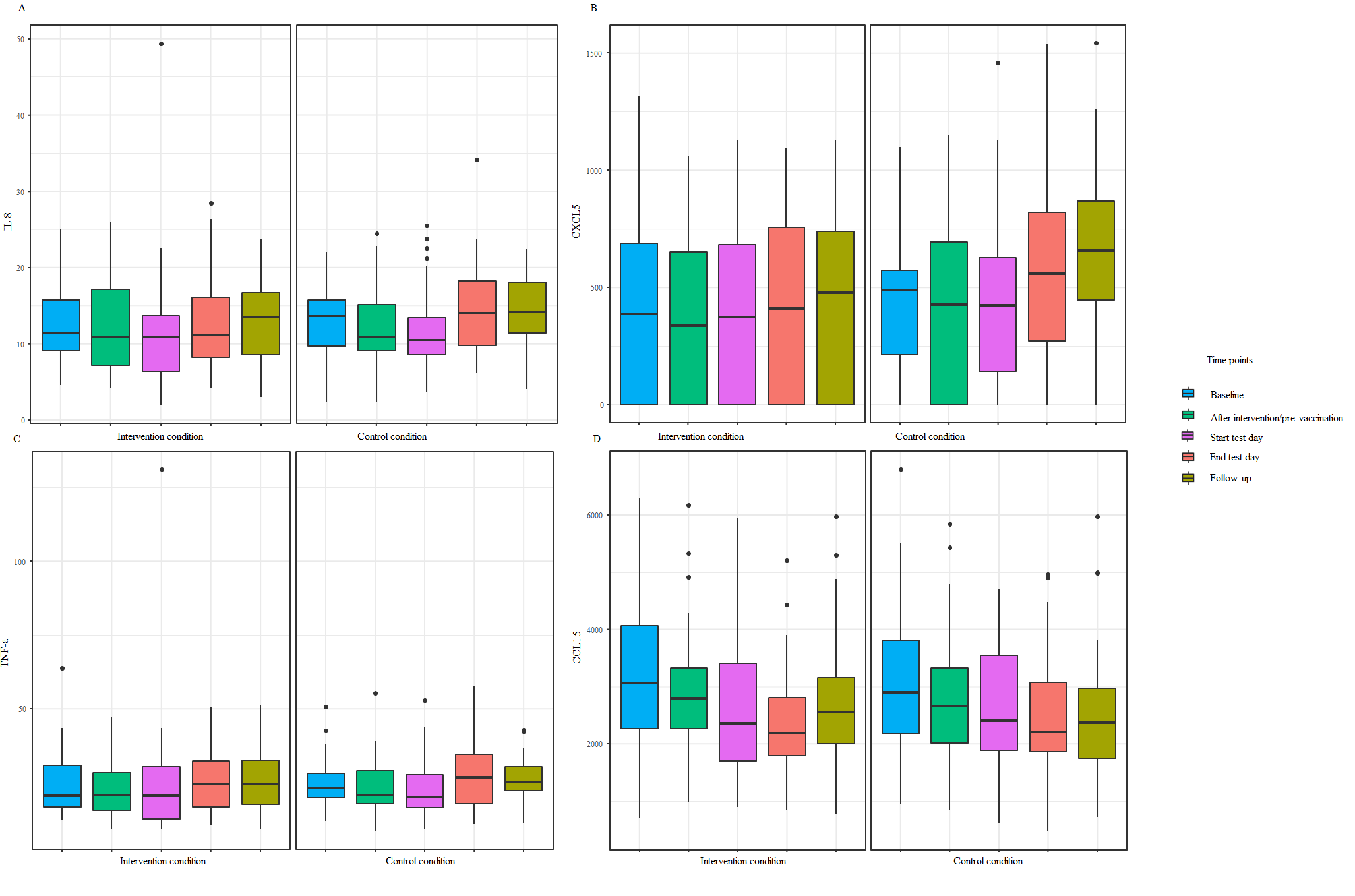

Supplement: Multimedia Appendix 5 [file jmir_v22i7e14861_app5.png]

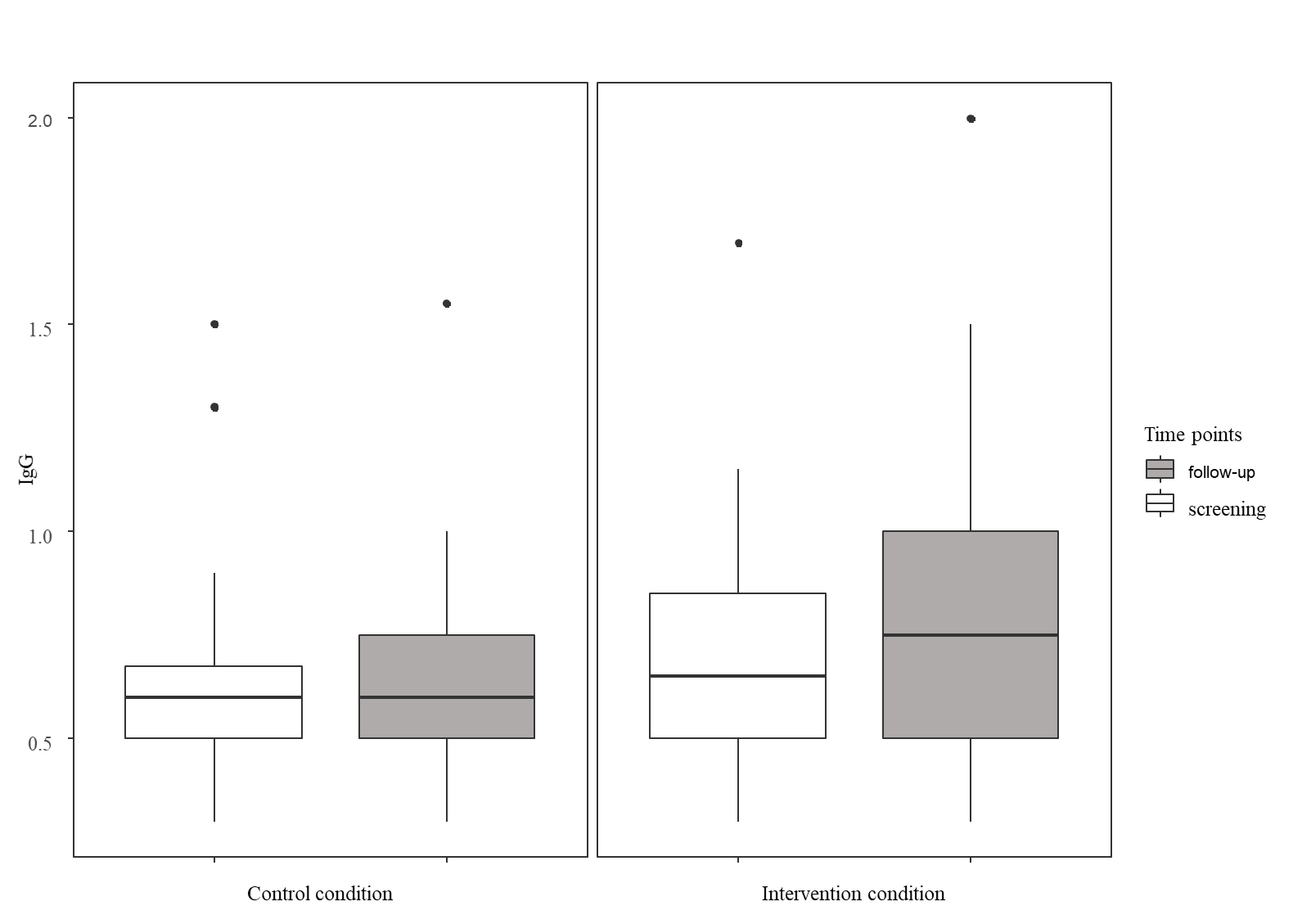

Supplement: Multimedia Appendix 6 [file jmir_v22i7e14861_app6.png]
