# Supplementary material for: An Internet-Based Psychological Intervention With a Serious Game to Improve Vitality, Psychological and Physical Condition, and Immune Function in Healthy Male Adults: Randomized Controlled Trial
Source: J Med Internet Res. 2020 Jul 24;22(7):e14861. doi: 10.2196/14861 (PMC7414409; doi:10.2196/14861)
Supplement: Multimedia Appendix 4 [file jmir_v22i7e14861_app4.docx]

|  | **Baseline** | **After intervention /pre-vaccination** | **Start test day** | **End test day** | **Follow-up** |
| --- | --- | --- | --- | --- | --- |
|  |  |  |  |  |  |
| HR  CC  IC | 68.3 (8.1)  66.6 (7.8) |  | 67.2 (9.1)  65.7 (9.0) | 63.5 (9.2)  64.1 (7.6) | 72.8 (13.4)  67.6 (9.4) |
| SC  CC  IC | 4.5 (2.3)  4.2 (2.2) |  | 3.8 (1.5)  4.7 (2.4) | 4.3 (1.7)  5.2 (3.7) | 4.3 (2.1)  4.9 (5.2) |
| HRV  CC  IC | 55.9 (38.4)  54.9 (25.1) |  | 54.4 (43.8)  55.0 (26.0) | 79.5 (79.5)  66.1 (35.0) | 44.1 (27.8)  58.7 (35.8) |
| Cortisol  CC  IC | 5.5 (4.1)  4.8 (1.9) | 6.2 (5.0)  6.0 (4.8) | 4.9 (2.6)  4.6 (1.3) | 7.9 (4.7)  6.7 (3.4) | 7.7 (6.7)  5.2 (1.6) |
| Alpha  Amylase  CC  IC | 2180.8 (1891.4)  2360.1 (2144.0) | 1248.1 (900.7)  1348.6 (744.4) | 1155.7 (791.6)  1211.2 (772.6) | 1810.6 (1740.5)  1709.1 (1149.0) | 1433.0 (1062.0)  1449.7 (1052.4) |

**Note.** CC = control condition, HR = heart rate, HRV = heart rate variability, IC = intervention condition, SC = Skin conductance. Cortisol is expressed in nanomoles per liter (nmol/L) and skin conductance is expressed in units per liter (U/L).
